# Energy Channel Coupling by Mid‐Trophic Level Fish Challenges the Landscape Theory for Food Web Architecture in a Large Tropical Lake Ecosystem

**DOI:** 10.1002/ece3.74036

**Published:** 2026-07-29

**Authors:** Madison F. Muehl, Jill A. Olin, James L. Keyombe, Josephine Y. Aller, Robert M. Cerrato

**Affiliations:** ^1^ School of Marine & Atmospheric Sciences Stony Brook University Stony Brook New York USA; ^2^ Department of Biological Sciences, Great Lakes Research Center Michigan Technological University Houghton Michigan USA; ^3^ Kenya Marine and Fisheries Research Institute Kisumu Kenya

**Keywords:** energy channels, fisheries, food webs, lake ecology, stable isotopes

## Abstract

Landscape theory for food web architecture (LTFWA) posits that the stability of food webs is supported by the coupling of fast pelagic and slow littoral energy channels mediated by large mobile consumers. Empirical tests of the LTFWA are limited, and support for the LTFWA varies among and within aquatic ecosystems. While the LTFWA is thought to be supported in large lake ecosystems, empirical studies have focused on temperate environments with little emphasis on large tropical lake ecosystems. Lake Turkana, a climatically sensitive East African Great Lake, is a highly resilient desert lake and an ideal model for examining how consumer fish species may mediate the coupling of fast and slow energy channels within food webs in a large tropical lake ecosystem. To empirically test the LTFWA, we explored the ecological niche characteristics of five important Lake Turkana fish species including *Alestes baremoze* , *Brachyalestes ferox*, *Hydrocynus forskahlii* , *Lates niloticus* , and *Oreochromis niloticus* across ontogeny and used a Bayesian isotope mixing model approach to identify which species play important roles in coupling the fast pelagic and slow littoral energy channels. We reveal that mid‐trophic level fish species like *A. baremoze* , *B. ferox* , and *O. niloticus* play important roles in coupling energy channels in Lake Turkana, subverting expectations that upper trophic level consumers *H. forskahlii* and *L. niloticus* would couple energy channels the most. We demonstrate that the energy coupling patterns proposed by the LTFWA are not ubiquitously supported across all large lake ecosystems, and small‐bodied mid‐trophic level fish species are vital for coupling energy channels in Lake Turkana. Thus, fisheries management in Lake Turkana should aim to sustain populations of mid‐trophic level species that couple pelagic and littoral energy channels via a multispecies approach that considers interspecific trophic interactions between targeted fish species.

## Graphical abstract

Lake Turkana, a large tropical East African Great Lake, is an ideal model for examining how consumer fish species may mediate the coupling of fast and slow energy channels within food webs. We explored the ecological niche dynamics of five important Lake Turkana fish species across ontogeny and used a Bayesian isotope mixing model approach to identify which species play important roles in coupling the fast pelagic and slow littoral energy channels.

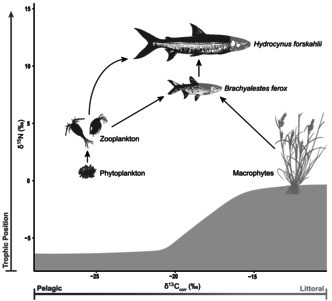

## 1 Introduction

Ecosystems are profoundly influenced by the flow of energy throughout their food webs. Food webs are supported by energy channels, or structures within ecosystems where low to mid‐trophic level consumers derive their energy from the same basal production sources. Rooney et al. (2006) underscored the critical link between food web stability, or the likelihood of the persistence of a set of interacting species, and the degree to which energy channels with distinct turnover rates are coupled via consumer species. Energy channel turnover rates are governed by both interaction strengths and life histories of species or functional groups within the food web (McCann et al. 1998; Rooney et al. 2006).

On average, “fast” pathways are comprised of stronger interaction strengths than “slow” pathways, and channels with high production to biomass ratios (e.g., pelagic phytoplankton production) exhibit short primary consumer generation times and fast turnover rates (Rooney et al. 2006). Landscape theory for food web architecture (LTFWA) posits that the coupling of these fast and slow energy channels occurs when high trophic level consumers feed on prey from multiple energy channels, as large upper trophic level consumers are thought to be more mobile than small lower trophic level consumers, moving between multiple habitats more often and consuming prey from multiple energy channels (McCann and Rooney 2009; Keppeler et al. 2021). Increased coupling of fast and slow energy channels is thought to contribute to the structural asymmetry of food webs that introduces local and non‐local stabilizing effects which are particularly important in systems with frequent environmental fluctuations (Rooney et al. 2006, 2008; Rooney and McCann 2012).

Empirical support for the LTFWA varies among ecosystems with strong support in marine and estuarine environments (Rooney et al. 2006; Rooney and McCann 2012; Potapov et al. 2019; Keppeler et al. 2021). However, the relationship between body size and trophic level is generally weaker in freshwater ecosystems compared to marine ecosystems, and empirical support of the LTFWA in freshwater ecosystems is more variable (Potapov et al. 2019; Maitland et al. 2024). Food webs in large lakes (> 500 km^2^) are thought to be structured similarly to marine ecosystems due to the presence of more geologically distinct habitats relative to small lakes and rivers (Potapov et al. 2019). However, empirical studies of the LTFWA in large lake ecosystems are concentrated in temperate regions with limited emphasis on low‐latitude large tropical lake ecosystems that are often important biodiversity hotspots and evolutionary hubs with high levels of endemism (Salzburger et al. 2014; Potapov et al. 2019; Maitland et al. 2024).

A quintessential example of one such large tropical lake ecosystem is Lake Turkana (~6405 km^2^), a highly dynamic East African Rift Valley desert lake shared by Kenya and Ethiopia. Due to its endorheic nature and high evaporation rates, Lake Turkana is often described as an “amplifier lake” as it has historically responded rapidly to even moderate climate variation with large changes in lake level that result in fluctuations in littoral and pelagic habitat distribution (Trauth et al. 2010). Despite this strong ecosystem response to climate variation, many fish genera currently present in Lake Turkana have persisted throughout a series of paleolakes that occupied the Turkana Basin from four million years ago to the onset of the modern Lake Turkana ~200,000 years ago (Stewart 2001; Prendergast and Beyin 2018).

Lake Turkana's aquatic food webs are additionally impacted by the growth of the lake's commercial fishery that has developed around the lake's highly productive littoral regions since the early 1960s. Ferguson's Gulf is the most productive fishing area of Lake Turkana, and fish species harvested from this community include *Alestes baremoze* , *Hydrocynus forskahlii* , *Lates niloticus* , and *Oreochromis niloticus* . There has also been recent support for the expansion of Lake Turkana's pelagic fishery to combat food scarcity in the surrounding region (Kolding et al. 2019) which would increase fishing effort targeting *A. baremoze* , *Brachyalestes ferox*, and *Synodontis schall* . Current fishing strategies have led to the harvest of primarily the largest fish in the targeted populations, not only contributing to changes in the growth and maturation dynamics of Lake Turkana's fish species (Muehl et al. 2025), but likely to changes in the inter‐ and intraspecific trophic interactions of fish. However, only a few studies have been conducted on the trophic interactions of Lake Turkana's fish species (Gownaris 2015; Gownaris et al. 2015).

Lake Turkana is a prime candidate for the empirical study of the LTFWA in large tropical lake ecosystems through relating body size, trophic position, and energy channel coupling. In this ecosystem, phytoplankton production represents a fast energy channel while C4‐marsh plant production represents a slow energy channel, and consumer fish species including *A. baremoze* , *B. ferox* , *H. forskahlii* , *L. niloticus* , and *O. niloticus* may play important roles in linking these two energy channels. Determining which consumer species strongly contribute to the coupling or decoupling of fast and slow energy channels provides vital insight into the ecological roles of Lake Turkana's fish species across life history with important implications for fisheries management, as future fishing‐induced changes in population structure may shift trophic interactions and energy flows.

Stable isotopes of nitrogen and carbon are used to estimate trophic positions of consumers (δ^15^N) and food web energy sources (δ^13^C), providing important information about trophic interactions and food web structure (Peterson and Fry 1987; Post 2002). Isotopic fractionation of ^15^N results in an increase in δ^15^N for each trophic level as ^15^N becomes enriched in a consumer relative to its prey (DeNiro and Epstein 1981; Minagawa and Wada 1984). In lake systems, δ^13^C can be used to assess the relative contributions of energy sources from littoral production (e.g., C4‐marsh plants) and pelagic production (e.g., phytoplankton). Littoral lake food webs are often enriched in ^13^C, corresponding to less negative δ^13^C values, and pelagic lake food webs are often less enriched in ^13^C, corresponding to more negative δ^13^C values (France 1995; Post 2002). Bayesian isotope mixing models can use these isotopic tracers to estimate the relative contributions of fast and slow energy channels to a consumer population as well as assess the drivers of shifts in these relative contributions across ontogeny (Moore and Semmens 2008; Stock et al. 2018).

In this study, we examine changes in energy channel coupling and trophic interactions across ontogeny for populations of five important Lake Turkana fish species including *A. baremoze* , *B. ferox* , *H. forskahlii* , *L. niloticus* , and *O. niloticus* . Specifically, we combine carbon and nitrogen stable isotope data with life history data (i.e., body size, maturity stage, and sex) to explore ecological niche variability and overlap between species and across ontogeny. We also employ a Bayesian isotope mixing model framework to identify which fish species play key roles in coupling fast and slow energy channels as well as evaluate how coupling or decoupling of energy channels changes across ontogeny to empirically test the LTFWA in a tropical large lake ecosystem.

Given conventional expectations that large lake ecosystems are structured similarly to marine ecosystems with mobile upper trophic level species coupling energy channels the most (Rooney et al. 2006; Potapov et al. 2019), we hypothesize that:

1. Large‐bodied, upper trophic level consumer species ( *H. forskahlii* and *L. niloticus* ) will exhibit higher degrees of “fast” pelagic and “slow” littoral energy channel coupling compared to low to mid‐trophic level consumer species ( *A. baremoze* , *B. ferox* , and *O. niloticus* ), supporting the LTFWA.
2. Energy channel coupling will increase with body size across all consumer species, indicated by a shift toward a balanced reliance on “fast” pelagic and “slow” littoral energy sources with increasing body size, supporting the LTFWA.

## 2 Methods

### 2.1 Sample Collection

**FIGURE 1:**
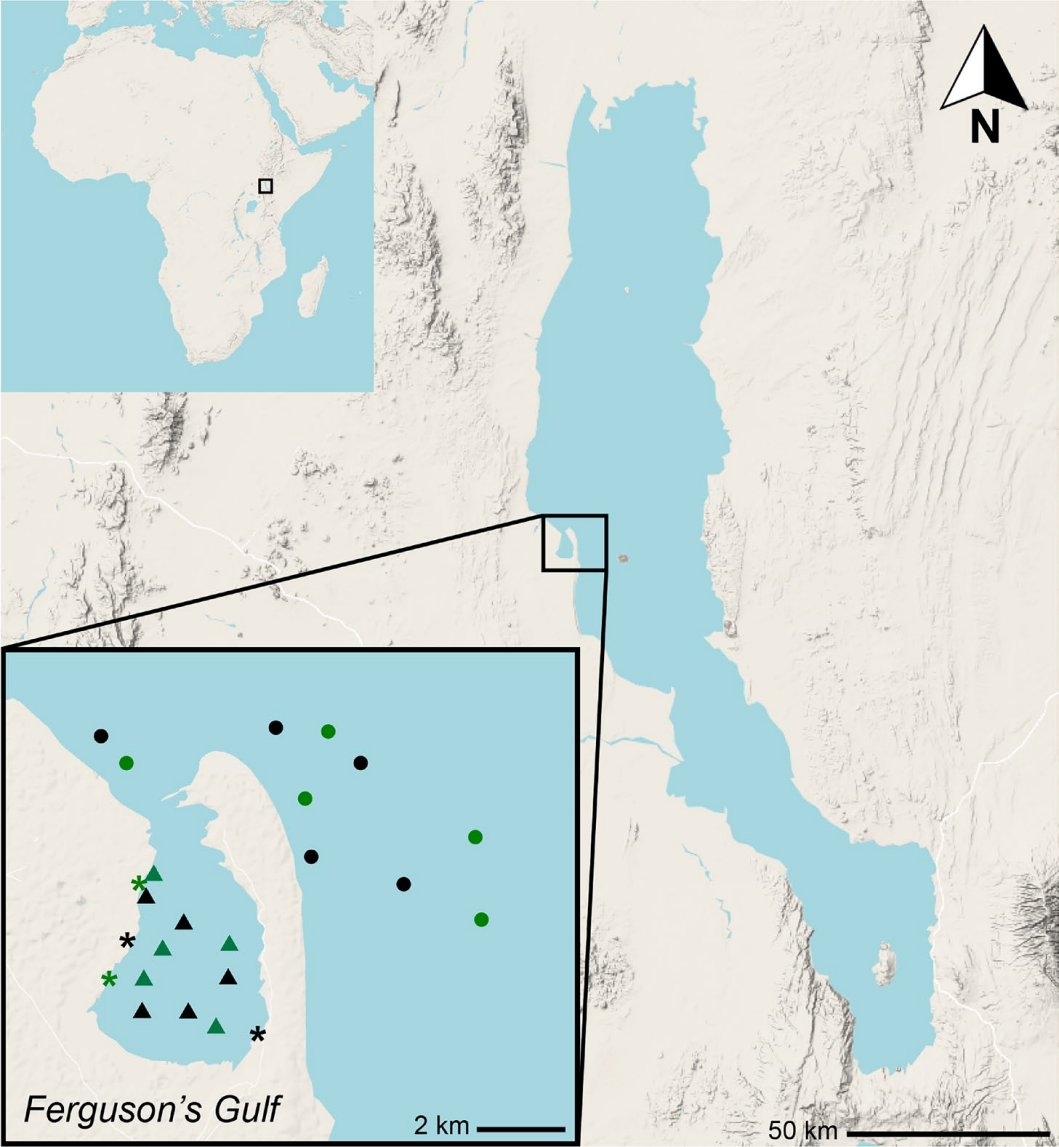
Map of Lake Turkana showing the western Ferguson's Gulf region (inset) with littoral and pelagic sampling sites indicated by triangles and circles, respectively. Littoral sampling sites from which C4‐macrophyte source samples were collected are indicated by asterisks. Sampling sites from 2022 are shown in black while sampling sites from 2024 are shown in green.

White muscle tissue was collected from five fish species, *Alestes baremoze* (*n* = 98), *Brachyalestes ferox* (*n* = 60), *Hydrocynus forskahlii* (*n* = 124), *Lates niloticus* (*n* = 88), and *Oreochromis niloticus* (*n* = 119), captured from five littoral and five pelagic sampling sites from the western marsh of Ferguson's Gulf to Central Island (Figure 1). Fish were captured during fisheries‐independent surveys in January 2022, 2023, and 2024 using surface‐set, unbaited scientific monofilament gillnets (68.6 m long, 3.1 m deep). Each gillnet contained 15 mesh panels (each 4.6 m long) with stretched mesh sizes of 2.5, 3.8, 5.1, 6.4, 7.6, 8.9, 10.1, 12.7, 14.0, 15.2, 16.5, 17.8, 20.3, and 21.6 cm. Survey sampling sites were randomly selected within the bounds of the established Ferguson's Gulf littoral fishery and the bounds of the currently expanding pelagic fishery. Two gillnets were deployed at each sampling site between 0600 and 1000 h and were retrieved after a soak time of 6 h.

Upon capture, fish were measured for total length (cm) and fork length (cm, when applicable) and weighed (kg). Sex, maturity status, and reproductive stage were determined for each fish via dissection during fieldwork and macroscopic gonad staging methods developed by Hopson (1982) and described in Table S1. Due to logistical constraints, sex and maturity data were not collected for *L. niloticus* . White muscle tissue samples (~2.0 g) were collected from each fish during fieldwork and immediately frozen at −20°C. Frozen samples were then cryogenically transported to Michigan Technological University, United States for stable isotope analyses.

Production sources were selected to represent two potential energy channels including a C4‐dominated littoral slow energy channel and a C3‐dominated pelagic fast energy channel. Lake Turkana's littoral areas are dominated by C4‐vegetation, specifically *Echinochloa stagnina* , with some C3‐marsh vegetation interspersed throughout. Macrophyte samples (*n* = 11) from *E. stagnina* , *Sporobolus marginatus*, *Typha* spp., and *Prosopis juliflora* were opportunistically collected from Lake Turkana littoral sites in January 2022 and 2024 (Figure 1).

Fish white muscle tissue typically integrates an isotopic signature over several months; thus, a series of samples composed of a mixture of phytoplankton and particulate organic matter samples (POM) (*n* = 45) were collected to capture a range of possible seasonal variability in phytoplankton/POM isotopic signatures given the fast biomass turnover of this source type. Logistical constraints due to the remote nature of Lake Turkana precluded the collection of a long time series of source isotopic signatures. Thus, phytoplankton/POM source samples representing dry season conditions were collected in January 2022 and during January, August, and September 2024, while phytoplankton/POM source samples representing wet season conditions were collected during March, April, and May 2024 at pelagic sites (Figure 1). We assume that the variability in isotopic signatures captured by sampling across wet and dry seasons is representative of the isotopic baseline variability incorporated into fish tissue from 2022 to 2024, and Wilcoxon rank‐sum test results revealed no significant difference between 2022 and 2024 for both δ^13^C (*W* = 109, *p* = 0.35) and δ^15^N (*W* = 96, *p* = 0.67) isotopic baselines. Phytoplankton/POM samples were collected via horizontal plankton tows followed by filtration to separate zooplankton from phytoplankton/POM by size fractionation. A subsample of each phytoplankton/POM sample was then microscopically examined to determine that secondary consumers like the large calanoid copepod, *Tropodiaptomus turkanae*, were not present in the samples.

### 2.2 Stable Isotope Analyses

Fish white muscle, C4‐marsh plants, and phytoplankton/POM were freeze‐dried and ground to homogenize each sample. Dried and ground samples were then weighed into tin capsules (500–1000 μg), and the relative abundances of carbon (^13^C/^12^C) and nitrogen (^15^N/^14^N) isotopes were analyzed using a Delta V Plus mass spectrometer coupled with an elemental analyzer in the Department of Biological Sciences at Michigan Technological University (Houghton, MI, USA). Results are expressed in standard delta (*δ*) notation, defined as parts per mille (‰) using Equation (1):

(1) $$\delta =\left[\left(\frac{R_{\text{sample}}}{R_{\text{standard}}}\right)-1\right]\times 10^{3}$$

where *R* is the heavy/light isotope ratio in the sample or the standard (Peterson and Fry 1987; Coplen 2011). Analytical precision was estimated relative to the standard deviation of internal standards run every 10 samples and was ±0.25‰ for δ^13^C and ±0.5‰ for δ^15^N. The ratio of total organic carbon to total organic nitrogen (C:N) was used to determine if high lipid content of samples may have negatively biased δ^13^C estimates, indicated by C:N ≥ 3.4 (Sweeting et al. 2006). As some fish samples had C:N ≥ 3.4, their isotopic values were lipid‐corrected using a non‐linear model by McConnaughey and McRoy (1979) modified by Logan et al. (2008) in Equation (2):

(2) $$\delta^{13}C_{\text{corr}}-\delta^{13}C=\frac{\left(a\times C:N+b\right)}{\left(C:N+c\right)}$$

where δ^13^C_corr_ represents lipid‐corrected carbon isotope values, δ^13^C represents the bulk tissue carbon isotope values, *a* is 7.415 ± −0.5576, *b* is −22.732 ± −1.5722, and *c* is 0.746 ± −0.5734.

### 2.3 Data Analyses

All statistical analyses were performed in R version 4.5.0 (R Core Team 2024). For each fish species, δ^13^C_corr_ and δ^15^N data were assessed for normality and homogeneity of variance with Shapiro–Wilk tests and Levene's tests, respectively. Results of Shapiro–Wilk and Levene's tests are presented in Tables S2 and S3, respectively. Due to non‐normality and heterogeneity of variances, univariate comparisons of both δ^13^C_corr_ and δ^15^N values between species and between maturity stages within each species were conducted using nonparametric Kruskal–Wallis tests followed by post hoc Dunn's tests with a Bonferroni correction (Dunn 1961) to adjust for multiple comparisons. Results of Kruskal–Wallis and Dunn's tests are presented in Tables S4 and S5, respectively. Since sets of δ^13^C_corr_ and δ^15^N values are derived from the same individual and are not truly independent, multivariate pairwise comparisons were also conducted using permutational multivariate analysis of variance (PERMANOVA) tests and subsequent pairwise comparisons with a Bonferroni correction (Dunn 1961) to adjust for multiple comparisons using the vegan package (Oksanen et al. 2013) and the pairwise.adonis() wrapper function from the package pairwiseAdonis (Martinez 2017) in R (R Core Team 2024).

Relationships between length (cm) and isotopic values were evaluated using linear regressions to preliminarily assess potential ontogenetic shifts in resource use (i.e., littoral or pelagic; δ^13^C_corr_) and trophic position (δ^15^N). A two‐endmember Bayesian isotope mixing model approach was then used to assess the relative contribution of littoral (i.e., C4‐dominant macrophytes) and pelagic (i.e., C3‐dominant phytoplankton) production sources to each fish population. Bayesian mixing models were implemented to estimate the relative contribution of production sources to each fish population, pooled across sampling years, while incorporating uncertainty using the MixSIAR package (Stock et al. 2018). Trophic enrichment factors for each fish species were selected based on the tissue type sampled (i.e., white muscle) and the feeding habits of each species based on a metanalysis by Stephens et al. (2023) (Table S6).

To assess the strongest predictors of relative resource use for each species, a suite of candidate mixing models with different covariate structures was run and compared to a null model (i.e., no predictors). For *A. baremoze* , *B. ferox* , *H. forskahlii* , and *O. niloticus* , four mixing models were run with covariates including length (continuous effect), sex (fixed effect), and maturity status (fixed effect). For *L. niloticus* , sex and maturity status data were not collected due to sampling limitations, and only one model was run with length (continuous effect) as a covariate. Mixing models were fit via Markov Chain Monte Carlo with three Markov chains, a chain length of 50,000 iterations, a burn‐in of 25,000, and a thinning interval of 25. Model convergence was assessed via Gelman‐Rubin and Geweke diagnostics (Geweke 1991; Gelman and Rubin 1992). Models were then compared via leave‐one‐out cross‐validation information criterion (LOOic) and Akaike weights to evaluate the strongest predictors of resource use variability for each fish species by testing each model on each data point after training on the remaining data points, with lower values indicating better predictive performance (Burnham and Anderson 2002; Vehtari et al. 2017).

Isotopic summary metrics were also calculated for each fish species and for each life history stage (i.e., immature and mature) with each species including δ^13^C_corr_ range (CR), δ^15^N range (NR), and standard ellipse area (SEA) representing a bivariate estimate of the size and shape of a consumer's isotopic niche based around 40% of the isotopic data (Jackson et al. 2011). CR provides insight into variability of consumer resource use while NR provides insight into consumer trophic variability (Layman et al. 2007). SEA estimates were calculated two ways including SEA and SEA estimated via a Bayesian approach to incorporate uncertainty (SEA_B_) (Jackson et al. 2011). SEA_B_ estimates of the 50% credible intervals were based on 10,000 posterior draws with a burn‐in of 1000. Isotopic niche overlap (%), or the probability that an individual from one species falls within the isotopic niche of another species, was calculated between species as well as between maturity stages within each species (Swanson et al. 2015). Isotopic niche overlap metrics were estimated over 10,000 iterations using the nicheROVER package in R (Swanson et al. 2015; Lysy et al. 2023).

## 3 Results

### 3.1 Isotopic Values and Interactions

**FIGURE 2:**
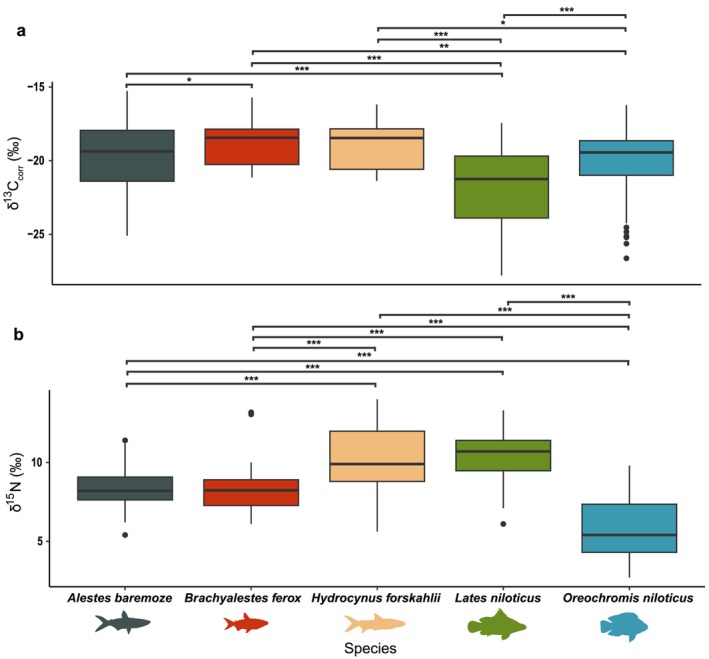
Boxplots of δ^13^C_corr_ (a) and δ^15^N (b) for *A. baremoze* (*n* = 98), *B. ferox* (*n* = 60), *H. forskahlii* (*n* = 124), *L. niloticus* (*n* = 88), and *O. niloticus* (*n* = 119). Boxplot elements include the minimum, 25th percentile, median, 75th percentile, and the maximum. Significant differences between species are indicated by the brackets, where *p ≤ 0.05, **p ≤ 0.01, and ***p ≤ 0.001. Corresponding Kruskal–Wallis and Dunn's test results are described in Tables S4 and S5, respectively.

**FIGURE 3:**
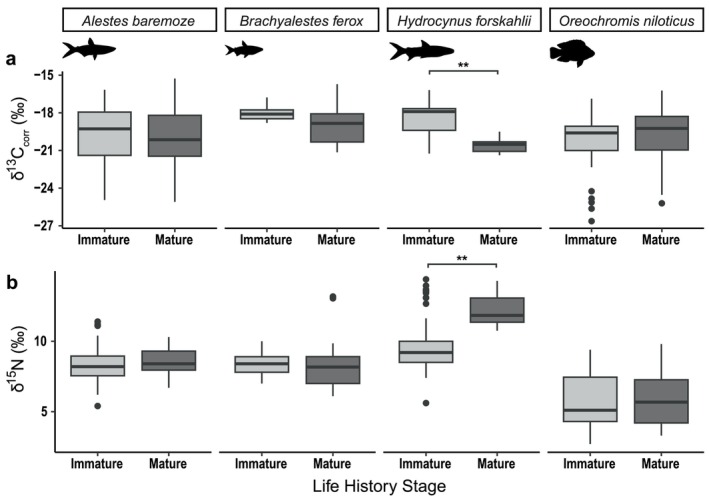
Boxplots of δ^13^C_corr_ (a) and δ^15^N (b) for *A. baremoze* (*n* = 98), *B. ferox* (*n* = 60), *H. forskahlii* (*n* = 124), and *O. niloticus* (*n* = 119) by maturity stage (immature and mature). Boxplot elements include the minimum, 25th percentile, median, 75th percentile, and the maximum. Significant differences between maturity stages are indicated by the brackets, where **p ≤ 0.01. Corresponding Kruskal–Wallis and Dunn's test results are described in Tables S4 and S5, respectively.

**FIGURE 4:**
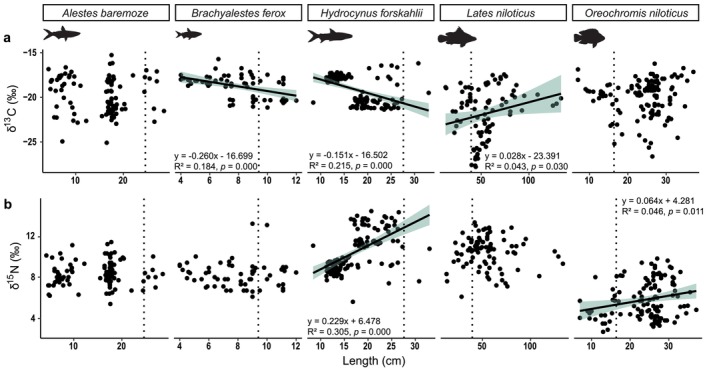
Linear regressions showing relationships between length (cm) and δ^13^C_corr_ (a) and δ^15^N (b) for *A. baremoze* (*n* = 98, FL), *B. ferox* (*n* = 60, FL), *H. forskahlii* (*n* = 124, FL), *L. niloticus* (*n* = 88, TL), and *O. niloticus* (*n* = 119, TL). Significant relationships are indicated by the black line with 95% confidence intervals in blue. Length at maturity (*L_mat_*; Muehl et al. 2025) is indicated by the dotted line.

Mean values and ranges of δ^13^C_corr_ and δ^15^N for each species and maturity stages are presented in Table 1. Univariate pairwise comparisons indicated significant differences in δ^13^C_corr_ between *A. baremoze* and *B. ferox* , *B. ferox* and *O. niloticus* , *H. forskahlii* and *O. niloticus* , and *L. niloticus* and all other species (Table S5, Figure 2a). All species exhibited significant differences in δ^15^N except *A. baremoze* and *B. ferox* and *H. forskahlii* and *L. niloticus* (Table S5, Figure 2b). Multivariate pairwise comparisons indicated significant differences in δ^13^C_corr_ and δ^15^N between all species (Table 2a). It is important to note that the significance detected for *B. ferox* and *H. forskahlii* may reflect a combination of centroid and dispersion due to the bimodal nature of the data. The results of additional comparisons between maturity stages of different species are outlined in Figure 3 and Table S5 for univariate pairwise comparisons and Table 2b for multivariate pairwise comparisons. Linear regression analyses showed significant relationships between δ^13^C_corr_ and length (cm) for *B. ferox* , *H. forskahlii* , and *L. niloticus* (Figure 4a) as well as between δ^15^N and length (cm) for *H. forskahlii* and *O. niloticus* (Figure 4b).

**TABLE 1:** Summary of length (cm), stable isotope values (‰), and elemental ratios from white muscle tissue samples of each species' life history stage (immature or mature).

Species	*n*	Length (cm)	δ^13^C_corr_ (‰)	δ^15^N (‰)	C:N
*Alestes baremoze*	98	14.9 ± 6.1 (4.3–28.5)	−19.8 ± 2.1 (−25.1 to −15.3)	8.4 ± 1.2 (5.4–11.4)	3.9 ± 0.9 (2.9–9.7)
Immature	79	13.4 ± 5.4 (4.3–25.5)	−19.7 ± 2.0 (−24.9 to −16.2)	8.3 ± 1.2 (5.4–11.4)	3.9 ± 0.9 (2.9–9.7)
Mature	19	21.2 ± 4.5 (16.4–28.5)	−20.1 ± 2.4 (−25.1 to −15.3)	8.6 ± 1.0 (6.7–10.3)	3.7 ± 0.6 (2.9–5.2)
*Brachyalestes ferox*	60	7.9 ± 2.2 (4.0–12.0)	−18.8 ± 1.3 (−21.1 to −15.7)	8.2 ± 1.3 (6.1–13.2)	3.7 ± 0.4 (3.1–4.5)
Immature	21	5.7 ± 1.4 (4.0–8.8)	−18.1 ± 0.5 (−18.8 to −16.8)	8.3 ± 0.9 (7.0–10.0)	3.6 ± 0.3 (3.2–4.4)
Mature	39	9.1 ± 1.5 (6.4–12.0)	−19.1 ± 1.4 (−21.1 to −15.7)	8.2 ± 1.5 (6.1–13.2)	3.7 ± 0.4 (3.1–4.5)
*Hydrocynus forskahlii*	124	17.6 ± 4.8 (8.4–33.0)	−19.2 ± 1.6 (−21.4 to −16.2)	10.5 ± 2.0 (5.6–14.4)	3.5 ± 0.4 (3.1–4.1)
Immature	85	15.7 ± 4.1 (8.4–30.8)	−18.5 ± 1.4 (−21.2 to −16.2)	9.7 ± 1.8 (5.6–14.4)	3.7 ± 0.3 (3.1–4.1)
Mature	39	21.8 ± 3.5 (17.2–33.0)	−20.6 ± 0.4 (−21.4 to −19.5)	12.2 ± 1.0 (10.8–14.3)	3.2 ± 0.2 (3.1–3.9)
*Lates niloticus*	88	54.7 ± 23.2 (12.8–133.5)	−21.8 ± 2.8 (−27.8 to −17.4)	10.4 ± 1.5 (6.1–13.3)	3.2 ± 0.4 (2.9–6.5)
*Oreochromis niloticus*	119	24.0 ± 6.6 (7.0–37.1)	−20.0 ± 2.1 (−26.6 to −16.2)	5.8 ± 1.8 (2.7–9.8)	3.3 ± 0.3 (2.9–4.1)
Immature	43	19.7 ± 7.0 (7.0–31.0)	−20.3 ± 2.2 (−26.6 to −16.9)	5.8 ± 1.9 (2.7–9.4)	3.3 ± 0.2 (3.0–3.9)
Mature	76	26.4 ± 5.0 (15.5–37.1)	−19.8 ± 2.0 (−25.2 to −16.2)	5.8 ± 1.8 (3.3–9.8)	3.3 ± 0.3 (2.9–4.1)

*Note:* Data are expressed as mean ± 1 SD with the range in parentheses. Fork lengths are presented for *A. baremoze* , *B. ferox* , and *H. forskahlii* while total lengths are presented for *L. niloticus* and *O. niloticus* .

**TABLE 2:** Summary of pseudo‐*F* statistics from pairwise multivariate PERMANOVA comparison of δ^13^C_corr_ and δ^15^N between species (a) and between species by life history stage (b).

a	*Ab*	*Bf*	*Hf*	*Ln*	*On*
*Ab*		7.90	45.34	49.87	52.16
*Bf*			40.40	66.91	47.41
*Hf*				45.56	199.22
*Ln*					142.17
*On*					

b		*Ab*		*Bf*		*Hf*		*On*	
		IMM	MAT	IMM	MAT	IMM	MAT	IMM	MAT
*Ab*	IMM		0.58	9.83	1.60	26.75	104.54	29.37	37.85
	MAT			10.94	2.49	10.75	56.65	13.10	16.50
*Bf*	IMM				5.19	8.63	257.25	26.70	25.79
	MAT					14.98	129.57	23.09	25.27
*Hf*	IMM						72.19	86.02	110.91
	MAT							168.51	212.57
*On*	IMM								1.14
	MAT								

*Note:* Larger pseudo‐*F* statistics indicate greater separation between groups. Statistical significance (*p*_adj_, or the probability of observing pseudo‐*F* given no difference between groups) is indicated by the color of box shading where white is no significance, yellow is *p*_adj_ ≤ 0.05, green is *p*_adj_ ≤ 0.01, and blue is *p*_adj_ ≤ 0.001.

### 3.2 Isotopic Variability and Isotopic Niche Overlap

**FIGURE 5:**
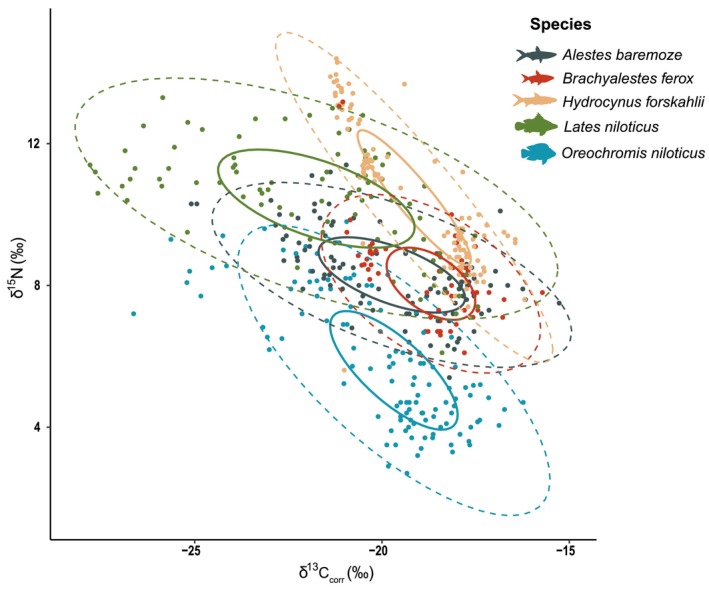
Isotopic biplots showing overlap between *A. baremoze* (*n* = 98), *B. ferox* (*n* = 60), *H. forskahlii* (*n* = 124), *L. niloticus* (*n* = 88), and *O. niloticus* (*n* = 119) with solid ellipses around 40% of the data and the dashed ellipses around 95% of the data.

**FIGURE 6:**
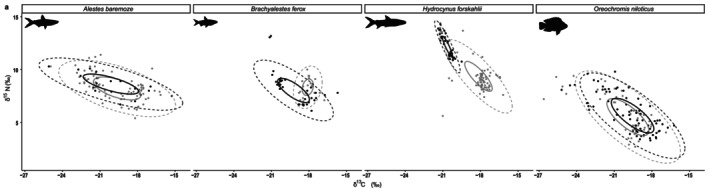
Isotopic biplots showing overlap between immature (light gray) and mature (dark gray) life history stages for *A. baremoze* (*n* = 98), *B. ferox* (*n* = 60), *H. forskahlii* (*n* = 124), and *O. niloticus* (*n* = 119) with the solid ellipses around 40% of the data and the dashed ellipses around 95% of the data.

*Oreochromis niloticus* and *L. niloticus* exhibited the largest CR while *H. forskahlii* exhibited the smallest CR (Table 3). Between life history stages within species, both mature *A. baremoze* and immature *O. niloticus* had the largest CR while mature *H. forskahlii* had the smallest CR (Table 3). *Hydrocynus forskahlii* exhibited the largest NR while *A. baremoze* exhibited the smallest NR (Table 3). Between life history stages within species, immature *H. forskahlii* exhibited the largest NR while immature *B. ferox* exhibited the smallest NR. Between species, SEA was greatest for *L. niloticus* and smallest for *B. ferox* (Table 3, Figure 5); and between life history stages within species, SEA was greatest for immature *O. niloticus* and smallest for mature *H. forskahlii* (Table 3, Figure 6). Estimates for SEA and SEA_B_ are consistent with one another and reflect similar results (Table 3).

**TABLE 3:** Summary of isotope niche metrics for each fish species by life history stage (immature or mature) including δ^13^C_corr_ (‰) range (CR), δ^15^N (‰) range (NR), standard ellipse area (SEA), and Bayesian estimates of standard ellipse area expressed as 50% credible intervals (SEA_B_).

Species	*n*	CR	NR	SEA (‰^2^)	SEA_B_ (‰^2^)
*Alestes baremoze*	98	9.8	6.0	6.2	5.8–6.6
Immature	79	8.8	6.0	6.3	5.8–6.7
Mature	19	9.8	3.6	5.5	4.7–6.5
*Brachyalestes ferox*	60	5.4	7.1	4.5	4.1–4.9
Immature	21	2.0	3.0	1.4	1.2–1.6
Mature	39	5.4	7.1	4.9	4.4–5.5
*Hydrocynus forskahlii*	124	5.2	8.8	5.3	5.0–5.7
Immature	85	5.1	8.8	5.4	5.0–5.8
Mature	39	1.9	3.5	0.6	0.6–0.8
*Lates niloticus*	88	10.4	7.2	11.2	10.4–12.0
*Oreochromis niloticus*	119	10.4	7.1	7.9	7.5–8.4
Immature	43	9.8	6.7	9.0	8.3–10.2
Mature	76	9.0	6.5	7.1	6.6–7.8

Isotopic overlap results between species and between maturity stages are described in Table 4. When comparing isotopic niche overlap between species, *A. baremoze* showed the highest probability of occurring within the isotopic niche of *L. niloticus* (87.68%) and the lowest probability of occurring within the isotopic niche of *H. forskahlii* (40.44%). *Brachyalestes ferox* showed the highest probability of occurring within the isotopic niche of *A. baremoze* (92.90%) and the lowest probability of occurring within the isotopic niche of *O. niloticus* (35.99%). *Hydrocynus forskahlii* showed the highest probability of occurring within the isotopic niche of *L. niloticus* (85.26%) and the lowest probability of occurring within the isotopic niche of *O. niloticus* (1.95%). *Lates niloticus* showed the highest probability of occurring within the isotopic niche of *A. baremoze* (55.61%) and the lowest probability of occurring within the isotopic niche of *O. niloticus* (19.00%). *Oreochromis niloticus* showed relatively low probability of occurring in the isotopic niches of all other species, with the highest probability being *A. baremoze* (25.83%) and the lowest probability being *H. forskahlii* (1.31%). Overall, *A. baremoze* , *B. ferox* , and *H. forskahlii* all showed high probability of occurring within the isotopic niche of *L. niloticus* , likely reflective of the large SEA_B_ of *L. niloticus* . However, *O. niloticus* exhibited the second largest SEA_B_ but did not show high probability of isotopic niche overlap with other species. This lack of isotopic overlap is likely due to the low δ^15^N values for *O. niloticus* , representative of lower trophic level feeding relative to the other four fish species.

**TABLE 4:** Estimates of isotopic overlap (%), or the probability that an individual of Species 1 would fall within the isotopic niche of Species 2, between species (a) and species by life history stage (b), *α* = 0.95.

a		Species 2				
		*Ab*	*Bf*	*Hf*	*Ln*	*On*
Species 1	*Ab*		75.12%	40.44%	87.68%	52.81%
	*Bf*	92.90%		67.52%	85.62%	35.99%
	*Hf*	45.55%	57.75%		85.26%	1.95%
	*Ln*	55.61%	37.60%	34.08%		19.00%
	*On*	25.83%	19.95%	1.31%	21.71%	

b			Species 2							
			*Ab*		*Bf*		*Hf*		*On*	
			IMM	MAT	IMM	MAT	IMM	MAT	IMM	MAT
Species 1	*Ab*	IMM		83.71%	27.36%	79.65%	48.39%	1.07%	49.32%	52.22%
		MAT	91.92%		25.11%	72.33%	44.96%	0.46%	46.88%	48.48%
	*Bf*	IMM	96.13%	87.51%		91.86%	96.01%	0.03%	13.80%	16.36%
		MAT	91.94%	77.42%	35.08%		63.76%	3.05%	40.60%	45.18%
	*Hf*	IMM	59.89%	44.02%	35.74%	57.82%		9.39%	3.52%	4.08%
		MAT	10.79%	3.58%	0.13%	28.01%	94.70%		0.05%	0.03%
	*On*	IMM	32.64%	17.73%	1.69%	19.62%	1.90%	0.01%		86.76%
		MAT	42.80%	22.15%	2.86%	31.06%	3.00%	0.01%	94.93%	

*Note:* Blue represents overlap values > 85% and orange represents overlap values < 15%.

When considering maturity stage, both immature and mature *A. baremoze* showed the highest probability of occurring within the isotopic niche of other mature and immature *A. baremoze* , respectively, and the second highest probability of occurring within the isotopic niche of mature *B. ferox* (Table 4b). This is also reflected in the high probability of both mature and immature *B. ferox* occurring within the isotopic niche of immature *A. baremoze* . Additionally, immature *B. ferox* showed particularly high probability (> 90%) of occurring within the isotopic niche of mature *B. ferox* and immature *H. forskahlii* (Table 4b). These probabilities are lower in mature *B. ferox* apart from a high probability of occurring within the isotopic niche of immature *A. baremoze* (91.94%), as previously mentioned (Table 4b). Multivariate pairwise comparisons of isotopic values between maturity stages of both species indicated that there is a significant difference in isotopic values between immature and mature *A. baremoze* and immature *B. ferox* , but not mature *B. ferox* (Table 2b). Univariate and multivariate pairwise comparisons between immature and mature *B. ferox* indicated no significant difference in isotopic values (Tables 2b and S5). Mature *B. ferox* also showed a larger SEA_B_ relative to immature *B. ferox* , indicating a broadening of isotopic niche after maturation (Table 3, Figure 6).

Immature *H. forskahlii* showed moderate probability of occurring within the isotopic niches of immature and mature *A. baremoze* and *B. ferox* (Table 4b). Notably, immature *H. forskahlii* showed low probability of occurring within the isotopic niche of mature *H. forskahlii* (9.39%), reflective of the small SEA_B_ estimated in mature *H. forskahlii* (Table 3). Similarly, mature *H. forskahlii* showed low probability (< 15%) of occurring with the isotopic niches of all other species and maturity stages except other immature *H. forskahlii* (94.70%) (Table 4b). Both immature and mature *O. niloticus* exhibited relatively low probability of occurring within the isotopic niches of other species (Table 4b). Immature *O. niloticus* showed the highest probability of occurring within the isotopic niche of mature *O. niloticus* (86.76%) and vice versa (94.93%) (Table 4b). However, low overlap probabilities did not seem to be driven by small isotopic niche areas in immature or mature *O. niloticus* , as SEA_B_ was still relatively large for both maturity stages (Table 3, Figure 6).

### 3.3 Mixing Model Results

**FIGURE 7:**
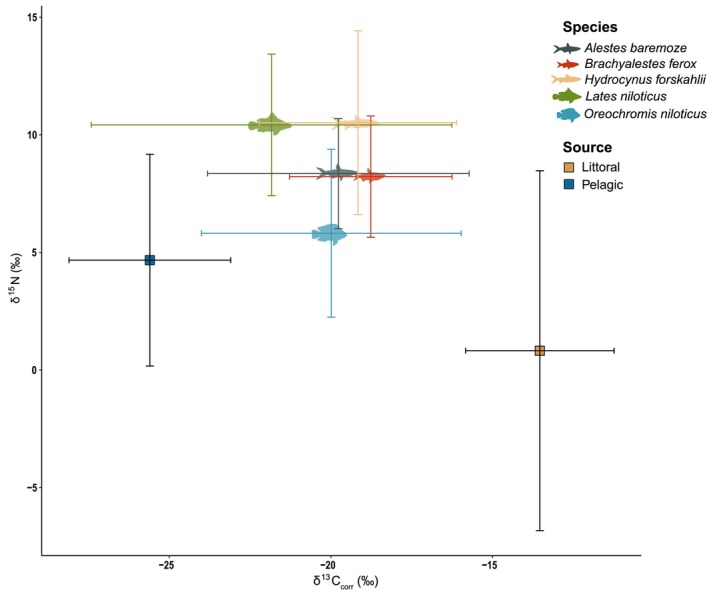
Isotopic biplot showing dietary mixing space for *A. baremoze* , *B. ferox* , *H. forskahlii* , *L. niloticus* , and *O. niloticus* with fast (i.e., pelagic phytoplankton) and slow (i.e., littoral C4marsh plant) energy channel endmembers.

**FIGURE 8:**
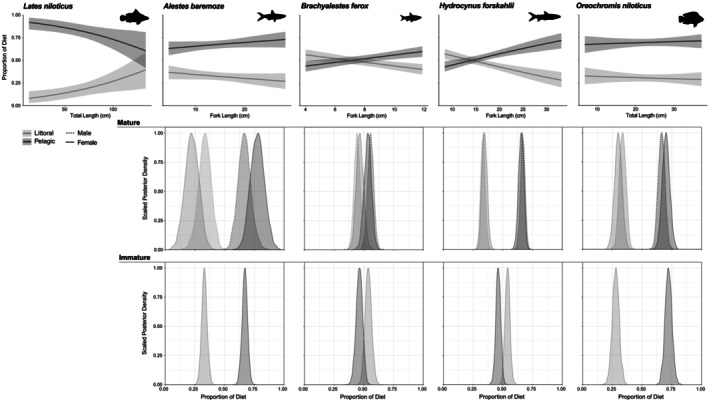
Scaled posterior density estimates of energy channel use for *A. baremoze* , *B. ferox* , *H. forskahlii* , *L. niloticus* , and *O. niloticus* by length (cm), sex, and maturity status.

All fish species analyzed fell within the littoral and pelagic δ^13^C_corr_ endmembers in mixing space (Figure 7). Bayesian isotope mixing model results indicated that pelagic production is the dominant pathway supporting *A. baremoze* with some reliance on littoral production, particularly at small sizes (Figure 8). Model posterior results indicated that production pathway reliance differs between mature male and female individuals, with females relying more heavily on the pelagic production pathway (Figure 8). However, model comparison results indicated that including the effect of sex (LOOic = 259.9, *w* = 0.268) did not significantly improve the model fit compared to the null model (LOOic = 259.5, *w* = 0.327) (Table 5).

**TABLE 5:** Comparison table of Bayesian isotope mixing models for each fish species that evaluate the drivers of the relative reliance on pelagic and littoral production pathways.

Species	Model	LOOic	SE LOOic	∆ LOOic	SE ∆LOOic	*w*
*Alestes baremoze*	Null	259.5	18.7	0.0	NA	0.327
	~Sex	259.9	18.7	0.4	4.1	0.268
	~Length	260.1	19.7	0.6	3.3	0.242
	~Maturity	260.9	18.7	1.4	2.2	0.162
*Brachyalestes ferox*	~Length	96.7	26.8	0.0	NA	0.650
	~Maturity	98.5	26.6	1.8	5.4	0.264
	~Sex	100.9	27.5	4.2	5.0	0.080
	Null	106.2	26.1	9.5	4.8	0.006
*Hydrocynus forskahlii*	~Sex	238.5	28.4	0.0	NA	1.000
	~Maturity	278.6	23.4	40.1	14.9	0.000
	~Length	311.1	22.2	72.6	22.1	0.000
	Null	323.1	17.1	84.6	20.8	0.000
*Lates niloticus*	~Length	302.8	14.3	0.0	NA	0.917
	Null	307.6	14.7	4.8	2.8	0.083
*Oreochromis niloticus*	Null	468.5	23.5	0.0	NA	0.430
	~Maturity	469.5	22.7	1.0	3.5	0.261
	~Sex	470.5	22.7	2.0	4.1	0.158
	~Length	470.6	23.2	2.1	1.2	0.151

*Note:* Leave‐one‐out cross validation scores (LOOic), the difference in LOOic scores from the most‐likely model (∆ LOOic), Akaike weights (*w*), and their associated standard errors (SE) are presented.

For *B. ferox* , Bayesian isotope mixing model results indicated that littoral production is the dominant pathway for individuals < 7.0 cm fork length, while pelagic production is the dominant pathway for individuals > 7.0 cm fork length (Figure 8). The most likely model included the effect of length (LOOic = 96.7, *w* = 0.650) (Table 5), revealing that *B. ferox* production pathway reliance is driven primarily by size regardless of sex or maturity status.

Similarly for *H. forskahlii* , Bayesian isotope mixing model results suggested that littoral production is the dominant pathway supporting small individuals < 14.0 cm fork length, while pelagic production is the dominant pathway supporting large individuals > 14.0 cm fork length (Figure 8). This revealed a decoupling of energy pathways at larger sizes with increased reliance on pelagic production (Figure 8). The most likely model included the effect of sex (LOOic = 238.5, *w* = 1.000) (Table 5), indicating that production pathway reliance differs between immature individuals as well as between mature males and females. This model outranked the model that included the effect of only maturity status (LOOic = 278.6, *w* = 0.000), indicating that sexes of adult *H. forskahlii* played an additional role in determining production pathway reliance.

For *L. niloticus* , Bayesian isotope mixing models revealed that pelagic production is the dominant pathway, particularly for small individuals as reliance on littoral production increases with body size (Figure 8). The most likely model included the effect of length (LOOic = 302.8, *w* = 0.917) (Table 5). It is important to note that sex and maturity data were not collected for *L. niloticus* , precluding the assessment of sex and maturity as drivers of production pathway reliance.

Bayesian isotope mixing models revealed that pelagic production is the dominant pathway supporting *O. niloticus* , with some reliance on littoral production (Figure 8). For *O. niloticus* , the null model (LOOic = 468.5, *w* = 0.430) was the most likely model, indicating that the relative contribution of energy pathways was not better explained by the addition of life history covariates including maturity, sex, or length (Table 5).

## 4 Discussion

The results of this study elucidate the roles of five fish species in the coupling and decoupling of Lake Turkana's fast and slow energy channels and how the degree of coupling changes across their life histories. Contrary to the expectations of the LTFWA, we determined that small mid‐trophic level fish species, including *A. baremoze* , *B. ferox* , and *O. niloticus* , couple fast and slow energy channels in Lake Turkana to a greater extent than large upper trophic level fish species, *H. forskahlii* and *L. niloticus* . Most notably, *B. ferox* plays a strong role in coupling Lake Turkana's fast and slow energy channels despite its small body size. These results also suggest that *B. ferox* relies on littoral production more than previously expected and may serve as an important conduit of C4‐marsh plant carbon to the large‐bodied species that prey on them. Thus, the previous classification of *Brachyalestes* spp. in Lake Turkana as “truly pelagic” (Kolding et al. 2019) may oversimplify their vital role in coupling Lake Turkana's littoral and pelagic energy channels. Both *A. baremoze* and *O. niloticus* rely primarily on Lake Turkana's fast energy channel but still derive some energy from the slow energy channel, and this remains relatively consistent across life history. The coupling of energy channels by mid trophic level fish species, particularly the small‐bodied *B. ferox* , contradicts our first hypothesis that the LTFWA would be supported in large lake ecosystems like Lake Turkana.

*Lates niloticus* was the only species that showed increased coupling at larger body sizes, likely due to its omnivorous feeding strategy and large isotopic niche area, consistent with previous research (Gownaris et al. 2015). Despite being an upper trophic level piscivore, *H. forskahlii* exhibited energy channel coupling at small body sizes below 14.0 cm fork length and decoupling at larger body sizes above 14.0 cm fork length, contrary to our second hypothesis. Decreased energy channel coupling with body size in *H. forskahlii* subverts the LTFWA expectation that large predatory species are more likely to couple fast and slow energy channels. The energy channel coupling patterns presented here differ from those that empirically support the LTWFA in Lake Michigan, a large temperate Laurentian Great Lake (Maitland et al. 2024). However, despite the general support for the LTFWA in Lake Michigan, some small‐bodied prey fish species couple energy channels more than previously expected (Maitland et al. 2024), and this phenomenon is more apparent in the tropical large lake ecosystem of Lake Turkana.

The decoupling of energy channels in *H. forskahlii* across ontogeny may be linked to an increase in trophic specialization after maturation, as mature *H. forskahlii* feed at a higher trophic position and display a more constricted isotopic niche (i.e., smaller SEA_B_) relative to immature *H. forskahlii* . Low overlap between mature and immature life history stages of *H. forskahlii* indicate a direct diet switch from littoral prey to pelagic prey, possibly reflecting movement offshore at the onset of maturation. These results ultimately support previous conclusions that *H. forskahlii* exhibit high trophic specialization (Gownaris et al. 2015) while providing a more nuanced understanding of how dietary specialization and habitat association may differ between life history stages. These data also suggest that Lake Turkana's littoral and pelagic gillnet fisheries target different life history stages of *H. forskahlii* with unique dietary strategies and niches, and the expansion of the offshore gillnet fishery would increase fishing pressure on mature *H. forskahlii* with more specialized diets.

The high probability that the isotopic niches of *A. baremoze* , *B. ferox* , and *H. forskahlii* would overlap with *L. niloticus* indicates that there is likely interspecific resource competition between *L. niloticus* and these three species, likely due to the generalist strategy of *L. niloticus* despite its primary reliance on the pelagic energy channel across the majority of its size range. Stomach content analyses by Hopson (1982) classified *L. niloticus* as piscivorous, feeding primarily on *O. niloticus* , *H. forskahlii* , and *Schilbe uranoscopus* . However, while some *L. niloticus* may have consumed small immature *H. forskahlii* , the isotopic results presented here indicate that many *L. niloticus* and *H. forskahlii* feed at the same trophic level. Thus, it is more likely that *L. niloticus* and *H. forskahlii* are competing for resources than *L. niloticus* feeding directly on *H. forskahlii* , and the reliance on multiple energy channels during opposite life history stages may help to alleviate some of this competition (Sánchez‐Hernández et al. 2019).

Lake Turkana's fishery is a “boom‐and‐bust” fishery, characterized by rapid increases in fisheries yield followed by abrupt decreases, driven by fluctuations in *O. niloticus* population size (Kolding 1993). Fishing‐induced population declines in species like *B. ferox* may result in a food web shift toward full reliance on fast energy channels that triggers consumer population overshoot after disturbance, increasing the time needed for consumer‐resource oscillatory dynamics to move back toward equilibrium (Rooney et al. 2006). The additional reliance on slow energy channels, governed by their weak interaction strengths, dampens consumer‐resource population oscillations (McCann et al. 1998; Post et al. 2000; Rooney et al. 2006). Thus, the presence of species that take advantage of both fast and slow energy channels is vital for supporting fast but stable consumer population recovery with reduced population overshoot after disturbance (Post et al. 2000; Emmerson and Yearsley 2004; Rooney et al. 2006). Dampened oscillatory population dynamics of mid‐trophic level prey species like *B. ferox* via the coupling of “fast” pelagic and “slow” littoral energy channels may introduce further stabilizing effects for predator species like *H. forskahlii* that exhibit more specialized diets.

The results of this empirical study of the LTFWA in Lake Turkana demonstrate that the LTFWA is not universally supported in all large lake systems, and the factors that govern energy channel coupling in Lake Turkana are more complex than mobility and body size alone. It is also important to note that weak but highly variable trophic interactions play an important role in maintaining community structure over space and time through the dampening of predator–prey population oscillations (Berlow 1999; McCann 2000). Future research should examine the spatiotemporal variance of Lake Turkana's trophic interactions to assess whether their strengths are universal or context dependent. Knowledge of the trophic interactions that govern energy flows and the identification of key fish species that couple Lake Turkana's fast and slow energy channels, presented here, ultimately increases our capacity for ecosystem‐based fisheries management that prioritizes food web stability and considers which species may dampen the oscillations of Lake Turkana's ‘boom‐and‐bust’ fishery.

## Author Contributions

**Madison F. Muehl:** conceptualization (equal), data curation (lead), formal analysis (lead), funding acquisition (supporting), investigation (lead), methodology (lead), project administration (lead), visualization (lead), writing – original draft (lead), writing – review and editing (lead). **Jill A. Olin:** conceptualization (equal), data curation (equal), formal analysis (equal), funding acquisition (supporting), investigation (equal), methodology (equal), project administration (equal), writing – original draft (equal), writing – review and editing (equal). **James L. Keyombe:** conceptualization (equal), data curation (supporting), funding acquisition (supporting), investigation (equal), methodology (equal), project administration (equal), writing – review and editing (equal). **Josephine Y. Aller:** conceptualization (equal), funding acquisition (lead), investigation (equal), project administration (equal), writing – original draft (equal), writing – review and editing (equal). **Robert M. Cerrato:** conceptualization (equal), data curation (equal), formal analysis (equal), methodology (equal), writing – original draft (equal), writing – review and editing (equal).

## Funding

This work was supported by Stony Brook University School of Marine and Atmospheric Sciences, Seed Grant (SEED2022 Aller).

## Conflicts of Interest

The authors declare no conflicts of interest.

## Supporting information


**Table S1:** Descriptions of reproductive stages for macroscopic gonad staging methods developed for Lake Turkana's fish species by Hopson (1982). Fish with reproductive stages ≥ 3 are considered mature, while fish with reproductive stages < 3 are considered immature.
**Table S2:** Shapiro–Wilk test results for δ^13^C_corr_ (‰) and δ^15^N (‰) by species and maturity stage. Degrees of freedom (df), Shapiro–Wilk test statistic (*W*), and significance values (*p*) are presented.
**Table S3:** Levene's test results for δ^13^C_corr_ (‰) and δ^15^N (‰) by species and maturity stage when the center is equal to the mean and when the center is equal to the median. Levene's *F*‐statistic (*F*) and significance values (*p*) are presented.
**Table S4:** Kruskal–Wallis test results for δ^13^C_corr_ (‰) and δ^15^N (‰) by species and maturity stage. Degrees of freedom (df), Kruskal–Wallis test statistic (*H*), and significance values (*p*) are presented.
**Table S5:** Summary of *Z*‐value statistics from pairwise univariate Dunn's Test comparison of δ^13^C_corr_ (a) and δ^15^N (b) between species and between species by life history stage (immature or mature). Statistical significance (*p*_adj_, or the adjusted probability of observing the *Z*‐value given no difference between groups) is indicated by the color of box shading where white is no significance, yellow is *p*_adj_ ≤ 0.05, green is *p*_adj_ ≤ 0.01, and blue is *p*_adj_ ≤ 0.001.
**Table S6:** Mean trophic discrimination factors (Δ^13^C and Δ^15^N) and standard deviations (SD) for fish species.

## Data Availability

The data associated with this research are available on the Dryad Digital Repository at the following link: https://doi.org/10.5061/dryad.bnzs7h4r1.
