# Supplementary material for: Energy Channel Coupling by Mid‐Trophic Level Fish Challenges the Landscape Theory for Food Web Architecture in a Large Tropical Lake Ecosystem
Source: Ecol Evol. 2026 Jul 29;16(8):e74036. doi: 10.1002/ece3.74036 (PMC13420381; doi:10.1002/ece3.74036)
Supplement: Supplementary file 1 — Table S1: Descriptions of reproductive stages for macroscopic gonad staging methods developed for Lake Turkana's fish species by Hopson (1982). Fish with reproductive stages ≥ 3 are considered mature, while fish with reproductive stages < 3 are considered immature. Table S2: Shapiro–Wilk test results for δ13Ccorr (‰) and δ15N (‰) by species and maturity stage. Degrees of freedom (df), Shapiro–Wilk test statistic (W), and significance values (p) are presented. Table S3: Levene's test results for δ13Ccorr (‰) and δ15N (‰) by species and maturity stage when the center is equal to the mean and when the center is equal to the median. Levene's F‐statistic (F) and significance values (p) are presented. Table S4: Kruskal–Wallis test results for δ13Ccorr (‰) and δ15N (‰) by species and maturity stage. Degrees of freedom (df), Kruskal–Wallis test statistic (H), and significance values (p) are presented. Table S5: Summary of Z‐value statistics from pairwise univariate Dunn's Test comparison of δ13Ccorr (a) and δ15N (b) between species and between species by life history stage (immature or mature). Statistical significance (p adj, or the adjusted probability of observing the Z‐value given no difference between groups) is indicated by the color of box shading where white is no significance, yellow is p adj ≤ 0.05, green is p adj ≤ 0.01, and blue is p adj ≤ 0.001. Table S6: Mean trophic discrimination factors (Δ13C and Δ15N) and standard deviations (SD) for fish species. [file ECE3-16-e74036-s001.docx]

**Appendix**

Table A1: Descriptions of reproductive stages for macroscopic gonad staging methods developed for Lake Turkana’s fish species by Hopson (1982). Fish with reproductive stages ≥ 3 are considered mature, while fish with reproductive stages < 3 are considered immature.

| **Maturity** | **Stage** | **Description** |
| --- | --- | --- |
| Immature | 0 | Undeveloped or very small gonads |
|  | 1 | Gonads are less than half the length of the ventral cavity |
|  | 2 | Developing gonads about half the length of the ventral cavity |
| Mature | 3 | Gonads are two-thirds the length of their ventral cavity |
|  | 4 | Gravid or have gonads filling the ventral cavity |
|  | 5 | Spent, gonads appearing shrunken or regressed in ventral cavity |

Table A2: Shapiro-Wilk test results for δ^13^C_corr_ (‰) and δ^15^N (‰) by species and maturity stage. Degrees of freedom (*df*), Shapiro-Wilk test statistic (*W*), and significance values (*p*) are presented.

|  |  | δ^13^C_corr_ (‰) | | δ^15^N (‰) | |
| --- | --- | --- | --- | --- | --- |
| Species | *df* | *W* | Sig. (*p*) | *W* | Sig. (*p*) |
| *Alestes baremoze* | 97 | 0.96 | 0.00 | 0.98 | 0.31 |
| Immature | 78 | 0.94 | 0.00 | 0.98 | 0.25 |
| Mature | 18 | 0.98 | 0.88 | 0.97 | 0.81 |
| *Brachyalestes ferox* | 59 | 0.92 | 0.00 | 0.87 | 0.00 |
| Immature | 20 | 0.95 | 0.39 | 0.96 | 0.46 |
| Mature | 38 | 0.91 | 0.00 | 0.84 | 0.00 |
| *Hydrocynus forskahlii* | 123 | 0.86 | 0.00 | 0.93 | 0.00 |
| Immature | 84 | 0.81 | 0.00 | 0.86 | 0.00 |
| Mature | 38 | 0.95 | 0.06 | 0.91 | 0.00 |
| *Lates niloticus* | 87 | 0.95 | 0.00 | 0.97 | 0.04 |
| *Oreochromis niloticus* | 118 | 0.93 | 0.00 | 0.94 | 0.00 |
| Immature | 42 | 0.87 | 0.00 | 0.93 | 0.01 |
| Mature | 75 | 0.95 | 0.01 | 0.94 | 0.00 |

Table A3: Levene’s test results for δ^13^C_corr_ (‰) and δ^15^N (‰) by species and maturity stage when the center is equal to the mean and when the center is equal to the median. Levene’s F-statistic (*F*) and significance values (*p*) are presented.

|  |  | **δ^13^C_corr_ (‰)** | | **δ^15^N (‰)** | |
| --- | --- | --- | --- | --- | --- |
|  | Center | *F* | Sig. (*p*) | *F* | Sig. (*p*) |
| By Species | Mean | 17.18 | 0.00 | 17.44 | 0.00 |
|  | Median | 12.53 | 0.00 | 13.55 | 0.00 |
| By Maturity | Mean | 14.47 | 0.00 | 6.27 | 0.00 |
|  | Median | 9.92 | 0.00 | 4.30 | 0.00 |

Table A4: Kruskal-Wallis test results for δ^13^C_corr_ (‰) and δ^15^N (‰) by species and maturity stage. Degrees of freedom (*df*), Kruskal-Wallis test statistic (*H*), and significance values (*p*) are presented.

|  |  | **δ^13^C_corr_ (‰)** | | **δ^15^N (‰)** | |
| --- | --- | --- | --- | --- | --- |
|  | *df* | *H* | Sig. (*p*) | H | Sig. (*p*) |
| By Species | 4 | 70.07 | 0.00 | 272.53 | 0.00 |
| By Maturity | 7 | 75.61 | 0.00 | 232.84 | 0.00 |

Table A5: Summary of *Z*-value statistics from pairwise univariate Dunn’s Test comparison of δ^13^C_corr_ (a) and δ^15^N (b) between species and between species by life history stage (immature or mature). Statistical significance (*p*_adj_, or the adjusted probability of observing the Z-value given no difference between groups) is indicated by the color of box shading where white is no significance, yellow is *p*_adj_ ≤ 0.05, green is *p*_adj_ ≤ 0.01, and blue is *p*_adj_ ≤ 0.001.

**a**

| δ^13^C_corr_ (‰) | *Ab* | *Bf* | *Hf* | *Ln* | *On* |
| --- | --- | --- | --- | --- | --- |
| *Ab* |  | 3.16 | 2.42 | -4.61 | -0.43 |
| *Bf* |  |  | -1.21 | -7.14 | -3.64 |
| *Hf* |  |  |  | -7.20 | -3.00 |
| *Ln* |  |  |  |  | 4.41 |
| *On* |  |  |  |  |  |

| δ^15^N (‰) | *Ab* | *Bf* | *Hf* | *Ln* | *On* |
| --- | --- | --- | --- | --- | --- |
| *Ab* |  | -0.50 | 6.96 | 6.58 | -6.61 |
| *Bf* |  |  | 6.50 | 6.26 | -5.18 |
| *Hf* |  |  |  | 0.19 | -14.4 |
| *Ln* |  |  |  |  | -13.3 |
| *On* |  |  |  |  |  |

| δ^13^C_corr_ (‰) | | *Ab* | | *Bf* | | *Hf* | | *On* | |
| --- | --- | --- | --- | --- | --- | --- | --- | --- | --- |
|  |  | IMM | MAT | IMM | MAT | IMM | MAT | IMM | MAT |
| *Ab* | IMM |  | -0.78 | 3.64 | 1.40 | 4.61 | -3.13 | -1.37 | -0.08 |
|  | MAT |  |  | 3.45 | 1.69 | 3.62 | -1.48 | -0.22 | 0.72 |
| *Bf* | IMM |  |  |  | -2.29 | -0.71 | -5.56 | -4.33 | -3.68 |
|  | MAT |  |  |  |  | 2.31 | -3.92 | -2.41 | -1.46 |
| *Hf* | IMM |  |  |  |  |  | -6.89 | -5.23 | -4.64 |
|  | MAT |  |  |  |  |  |  | 1.60 | 3.04 |
| *On* | IMM |  |  |  |  |  |  |  | 1.29 |
|  | MAT |  |  |  |  |  |  |  |  |

**b**

| δ^15^N (‰) | | *Ab* | | *Bf* | | *Hf* | | *On* | |
| --- | --- | --- | --- | --- | --- | --- | --- | --- | --- |
|  |  | IMM | MAT | IMM | MAT | IMM | MAT | IMM | MAT |
| *Ab* | IMM |  | 0.73 | 0.00 | -0.43 | 4.32 | 7.31 | -5.14 | -6.07 |
|  | MAT |  |  | -0.59 | -0.97 | 1.92 | 4.45 | -4.22 | -4.53 |
| *Bf* | IMM |  |  |  | -0.31 | 2.77 | 5.29 | -3.66 | -3.96 |
|  | MAT |  |  |  |  | 3.93 | 6.70 | -4.02 | -4.52 |
| *Hf* | IMM |  |  |  |  |  | 3.91 | -8.82 | -10.50 |
|  | MAT |  |  |  |  |  |  | -10.90 | -12.20 |
| *On* | IMM |  |  |  |  |  |  |  | 0.00 |
|  | MAT |  |  |  |  |  |  |  |  |

Table A6: Mean trophic discrimination factors (Δ^13^C and Δ^15^N) and standard deviations (SD) for fish species.

|  | **Δ^13^C (‰)** | | **Δ^15^N (‰)** | |
| --- | --- | --- | --- | --- |
|  | Mean | SD | Mean | SD |
| *A. baremoze* | 1.9 | 0.3 | 3.3 | 0.2 |
| *B. ferox* | 1.0 | 0.8 | 2.9 | 0.5 |
| *H. forskahlii* | 1.0 | 0.8 | 2.9 | 0.5 |
| *L. niloticus* | 1.7 | 0.4 | 4.3 | 0.4 |
| *O. niloticus* | 1.9 | 0.3 | 2.0 | 0.5 |
